# Comparison of growth dynamics in different types of MPS: an attempt to explain the causes

**DOI:** 10.1186/s13023-022-02486-4

**Published:** 2022-09-05

**Authors:** Agnieszka Różdżyńska-Świątkowska, Anna Zielińska, Anna Tylki-Szymańska

**Affiliations:** 1 Anthropology Laboratory, Children’s Memorial Health Institute, Warsaw, Poland; 2 Department of Paediatrics, Nutrition and Metabolic Diseases, Children’s Memorial Health Institute, Warsaw, Poland

**Keywords:** Mucopolysaccharidoses, Lysosomal storage disorders, Hurler syndrome, Sheie syndrome, Sanfilippo syndrome, Hunter syndrome, Maroteaux–Lamy syndrome, Growth pattern, Growth charts

## Abstract

**Background:**

Mucopolysaccharidoses (MPS) are a group of lysosomal storage disorders caused by deficient activity of enzymes responsible for the catabolism of glycosaminoglycans (GAGs), resulting in progressive damage to various tissues and organs. Affected individuals present with skeletal deformities, bone growth impairment, joint stiffness and frequently mental retardation.

**Results:**

The objective of the study was to summarise over 30 years of observations of the growth dynamics in patients with different types of MPS, performed at the Children’s Memorial Health Institute (CMHI, Warsaw, Poland). A retrospective analysis of anthropometric data collected from 1989 to 2020 was performed for 195 patients with MPS I, MPS II, MPS III, MPS IVA and MPS VI. Mean values for birth body length were statistically significantly greater than in the general population. The mean z-scores for other MPS groups showed that until the 24th month of life, the growth pattern for all patients was similar, and the average z-scores for body height were greater than in reference charts. Afterwards, growth patterns began to differentiate for MPS groups.

**Conclusions:**

The long-term follow up showed that the growth pattern in patients with all types of mucopolysaccharidoses significantly deviates from the general population. Patients with MPS IVA had the most severe growth impairments compared to other patients in the study group. Neuropathic MPS I and II demonstrated severe growth impairments compared to other patients in this study. Patients with MPS III showed the mildest growth impairments compared to other MPS patients and reached the 3rd percentile last.

## Introduction

Mucopolysaccharidoses (MPSs) are a group of lysosomal storage disorders caused by deficient activity of enzymes responsible for the catabolism of glycosaminoglycans (GAGs), resulting in progressive damage to various tissues and organs. Affected individuals with MPS I, II, VI present with coarse facial features, short stature, skeletal deformities, joint stiffness and frequently mental retardation. Mucopolysaccharidosis type I (MPS I) is caused by deficient activity of alpha-L-iduronidase (IDUA; EC 3.2.1.76); it is divided into three subtypes based on the severity of symptoms: Hurler syndrome (severe, OMIM 607016), Hurler–Scheie syndrome (intermediate, OMIM 607015) and Scheie syndrome (attenuated, OMIM 607016). Mucopolysaccharidosis type II (MPS II, Hunter disease, OMIM 309900) is an X-linked recessive disorder caused by deficient activity of iduronate-2-sulphatase (IDS, EC 3.1.6.13). Mucopolysaccharidosis type III (MPS III, Sanfilippo syndrome) includes four types, depending on the deficient enzyme: heparan N-sulphatase (type A, OMIM 252900), alpha-N-acetylglucosaminidase (type B, OMIM 252920), acetyl CoA:alpha-glucosaminide acetyltransferase (type C, OMIM 252930) and N-acetylglucosamine 6-sulphatase (type D, OMIM 252940). Despite the deficiency in a different enzyme, the clinical picture of patients with MPS III is very similar; therefore, these four types are called Sanfilippo syndrome.

Mucopolysaccharidosis type IV includes two types depending on the deficient enzyme: galactosamine-6-sulfatase (MPS IVA, type A, Morquio syndrome, OMIM 253000) and beta-galactosidase (MPS IVB, GLB1; 611458, OMIM 253000). Mucopolysaccharidosis type VI (MPS VI, Maroteaux-Lamy syndrome, MPS VI, OMIM 253200) is caused by deficient activity of *N*-acetylgalactosamine-4-sulphatase (4-sulphatase, arylsulphatase B, ARSB, EC 3.1.6.12) [1]. Chuang et al. (cyt) illustrated the difference in GAGs cumulation in different type of MPSes and showed the relationships between GAGs and phenotype.

Human growth is a multi-factorial and complex process involving a physiological interplay between metabolic, endocrine and nutritional factors in a broader background of variation in genetic traits and environmental exposure. MPS diseases lead to a profound disruption in normal mechanisms of growth and development [2]. In our previous studies, we evaluated and compared growth patterns in patients with MPS I and MPS II [3–5], MPS IVA [6] and MPS VI [7]. We also evidenced that at the time of birth, many MPS patients are larger compared to the general population [4]. The purpose of this work was to summarise over 30 years of observations of the growth dynamics in patients with MPS, remaining under the care of the Children’s Memorial Health Institute (CMHI, Warsaw, Poland).

## Methods

### Study group

A a mix-longitudinal retrospective analysis of anthropometric data from 1989 to 2020 was performed at the Children’s Memorial Health Institute for patients with MPS I (males n = 18), MPS II (males n = 56), MPS III (n = 72; females = 33 and males = 39), MPS IVA (there was no data from patients with MPS B) (n = 20; females = 7; males = 13) and MPS VI (n = 29; females = 18, males = 11). The total number of patients was 195; they were aged from 3 months to 18 years. Total number of measurements was 743. Each patient was measured a few times (ranged from 1 to 14 times) while monitoring was performed. The interval between measurements ranged from 3 months to 10 years.All patients were born at term (prematurely born patients were excluded from the study). Diagnosis of MPS was confirmed by enzymatic and molecular analyses in all patients. All patients were of Polish origin. Only data before treatment were taken into consideration.

### Study design and treatments

Birth body length and weight were taken from the children’s health records, where possible. Next, mean birth body length and weight were calculated. Each patient was measured a few times (ranging from 1 to 10 times) during periodic assessments. All measurements were carried out in the Anthropology Laboratory at CMHL with professional equipment. A Wolański liberometer (an infantometer accurate to 1 mm) was used to measure the supine length of children under 3 years. A stadiometer (accurate to 1 mm) was used to measure the standing height of older children. In the case of a serious problem with standing, segmental height in the supine position was estimated.

### Data analysis

Statistical analysis was performed using Statistica, v.8 (StatSoft, Krakow, Poland). Shapiro–Wilk and Kołmogorov–Smirnov tests were employed to assess sample normality for each parameter. The significance level was assumed at 0.05. A two-tailed t-test was used to compare the mean body length and weight values at birth between MPS patients and Polish reference charts.

The data for the individual groups of children were divided into calendar age classes. The degree and direction of deviations of growth measurements in children with MPS were analysed using a data standardisation method, and calculated values were presented as z-scores. In addition, the growth trend for body height was evaluated using a straight-line regression model.

## Results

### Birth body length and weight

Mean values for birth body length and weight for all studied mucopolysaccharidoses were greater than in the general population (Table 1). For body length, the differences were statistically significant. MPS IV A mean birth’s length was the largest, but differences in comparison to other types of MPS were not statistically significant. Differences in birth body weight were significant only for girls with MPS III. For MPS I, II and IVA, we did not have enough data on boys to perform statistical analysis.

**Table 1:** Comparison of birth length, weight and head circumference among patients with MPS and healthy control

	Body length (cm)	*p* value	Body weight (kg)	*p* value	Occipital frontal circumference (cm)	*p* value
MPS I						
Boys	55.6 ± 3.45 (n = 18)	**0.01**	3.47 ± 0.61 (n = 18)	0.83	34.4 ± 1.57 (n = 11)	0.09
MPS II						
Boys	55.4 ± 2.68 (n = 47)	**0.01**	3.63 ± 0.56 (n = 56)	0.09	34.6 ± 1.69 (n = 34)	**0.02**
MPS III						
Girls	53.4 ± 4.0 (n = 26)	**0.01**	3.15 ± 0.623 (n = 29)	**0.04**	33.7 ± 1.6 (n = 27)	**0.02**
Boys	55.3 ± 2.84 (n = 31)	**0.01**	3.53 ± 0.42 (n = 35)	0.78	34.9 ± 1.86 (n = 27)	0.27
MPS IVA						
Boys	57.3 ± 3.34 (n = 10)	**0.01**	3.72 ± 0.46 (n = 14)	0.35	bd	
MPS VI						
Girls	53.8 ± 2.89 (n = 16)	**0.01**	3.62 ± 0.56 (n = 18)	0.09	bd	
Boys	55 ± 3.59 (n = 10)	**0.01**	3.82 ± 0.61 (n = 11)	0.11	bd	
Healthy controls						
Girls	51.3 ± 2.4		3.4 ± 0.5		34.5 ± 1.3	
Boys	52.2 ± 2.8		3.5 ± 0.6		35.3 ± 1.6	

Bold font indicates statistically significant values

### Tendency of growth

**Fig. 1:**
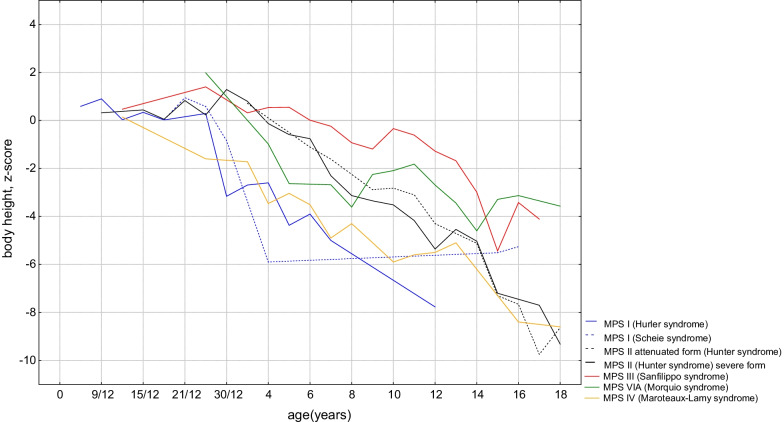
The standardized mean values for body height z-scores for MPS groups in calendar age classes

**Fig. 2:**
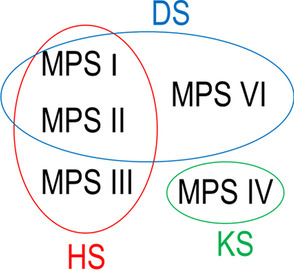
GAGs cumulated in different types of MPS. Red corresponds to heparan sulphate, green—keratan sulphate, blue—dermatan sulphate

Individual data for this study were standardised to show an actual degree and the direction of deviations. There were no differences in z-scores for body height in age groups between males and females among MPS I, MPS II, MPS III, MPS IVA and MPS VI patients; thus, both sex groups were analyzed together. From the very beginning, the mean z-scores for body height were smaller in comparison to the reference charts [8]. After birth, children with MPS IVA grew slowly, and reached their final height at approximately 8 years of age. For MPS VI, before the 5th year of life, the upward trend changed to a constant one, and the subsequent declines were not as sharp as in the first four years of life. The mean z-scores for other MPS groups (MPS I, II and III) showed that until the 24th month of life, the growth pattern for all patients was similar, and the average z-scores for body height were greater than the reference charts. Afterwards, growth patterns began to differentiate for individual groups. For Hurler syndrome, the body height below the 3rd percentile was noted from the 24th month of life, for patients with severe MPS II between the 6th and 7th year of life and for patients with attenuated MPS II between the 8th and 9th year. Patients with MPS III exceeded the 50th percentile by 6, then their growth was slower but exceeded the 3rd percentile by 14. For patients with MPS III and MPS VI, an acceleration of growing between 9 and 14 was observed, which could reflect puberty spurt (Figs. 1 and 2).

A linear regression model was used to show the directional changes in the growth of MPS patients. Because of insufficient suitability of data concerning MPS VI to model growth, the analysis only consisted of patients with types MPS I, MPS II, MPS III and MPS IVA. The above observation showed that growth in patients with MPS I, II and III could be divided into two periods. First, when the pace of development was faster, and the measurements were bigger than in the general population. And second, when the pace of development slowed down. This observation showed that the period of normal growth in patients with MPS III seems to be the longest, slightly shorter in patients with MPS II and the shortest in patients with MPS I. With regard to these differences, the MPS group was divided into two age groups: before the 3rd year of life and after the 3rd year of life. The straight-line regression model was made for each group separately to detect growth trends at different times in ontogenesis.

**Fig. 3:**
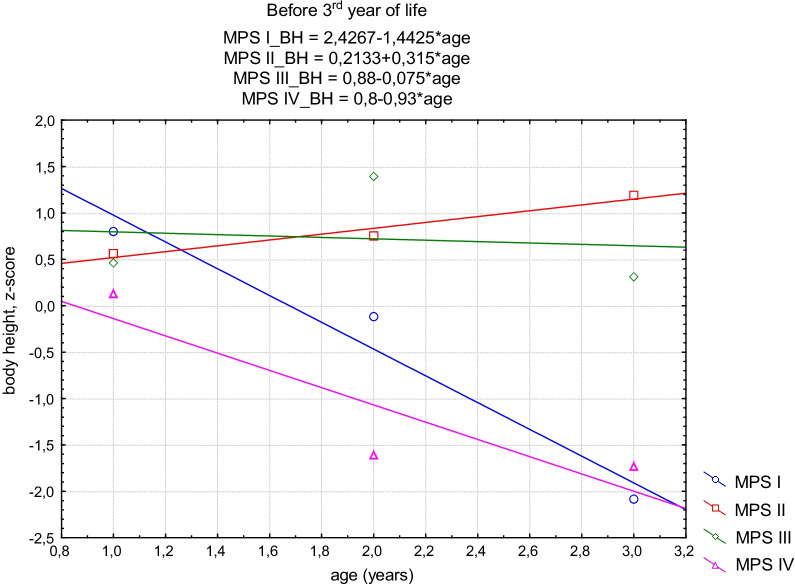
The straight-line regression model of standardized body height for all MPS groups before the 3rd year of life

**Fig. 4:**
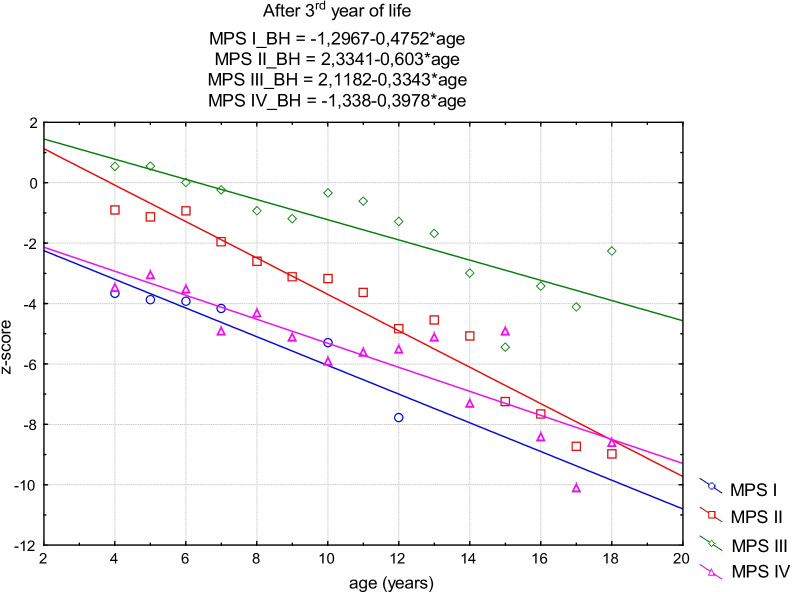
The straight-line regression model of standardized body height for all MPS groups after the 3rd year of life

The tendency of growth in the first period was negative but not statistically significant for MPS I (*p* = 0.13), MPS III (*p* = 0.92) and MPS IVA (*p* = 0.29). For MPS II, it was positive in the first period but also not statistically significant (*p* = 0.14). In the second period, the trend was negative and statistically significant for all MPS groups (MPS I, *p* = 0.01; MPS II, *p* = 0.01; MPS III, *p* = 0.01; MPS IVA, *p* = 0.02) (Figs. 3, 4).

## Discussion

‘Growth is a mirror of health’, James Tanner wrote these words, which have become fundamental to understanding the concept of auxology. For a rare disease, we could paraphrase them and say that ‘growth is a mirror of the severity of the disease’. In our previous studies, we evaluated and described growth in patients with MPS I, MPS II, MPS IVA and MPS VI [4–6, 9]. In this study, we attempt to answer the questions of the causes of differences in the growing process of MPS groups. In our cohort, mean values for birth length and weight were greater than in the general population, with statistically significant differences in birth length. This observation was congruent with further studies [4, 10, 11]. In our study, the birth length of MPS IVA patients was the largest in comparison to the healthy population and other types of MPS. However, the tendency for increased birth body length in patients with MPS IV was also observed in another study [12]. Patients with MPS IVA are longer at birth than children in the general population maybe because excessive laxity of the connective tissue allows the newborn to stretch out better when measured.

There is a scarcity of literature concerning the process of growth in children with different types of MPS diseases. Growing categorises clinical severity of the MPS disease. MPS IVA shows the most significant growth impairment. After birth, children with MSP IV grew slowly, and their final height corresponded to that of a 7-year-old child. The same observation was corroborated in previous publications [13–15]. A very similar trend of growth was observed in MPS VI patients. They were larger at birth and grew slower than their healthy peers. MPS VI patients fell down on percentile charts until about 8, and then the growth rate z-scores ranged between − 3 and − 4. The growth of patients with MPS I, II and III up to the 24th month of life was between 0 and 2 in body height z-scores. Afterwards, growth slowed down after the 2nd year of life for MPS I patients, around the 4th year of life for MPS II patients and around the 6th year of life for MPS III patients. After this period of intensive growth, in subsequent years, body height showed significantly lower values when compared with the reference charts. This trend was corroborated by earlier publications [10, 11, 16, 17]. The reasons for differences in growth dynamics between patients with different types of MPS are yet to be fully explained. One explanation is the accumulation dependency of DS. There is preclinical and clinical evidence of prenatal accumulation of GAGs in chondrocytes in MPS [2, 18–21]. The overgrowth in foetal and early postnatal life could be connected to HS acting as a coreceptor that binds to several proteins, including growth factors. Therefore, an increased level of HS might overstimulate axial bone growth in children with MPS I, II and III [22, 23]. This is contradicted by the fact that HS is not accumulated in MPS IVA, but they still are longer at birth than in the general population. At the same time, the accumulation of DS over time would cause inhibition in the analysis of MPS growth plates showing clusters of enlarged and GAG-containing cells that disrupt a columnar architecture of growth plate cartilage, presumably leading, in part, to abnormal bone growth [24]. Simonaro et al. suggested that the main tissue of these disorders is the cartilage rather than the bone itself. However, Hinek and Wilson [22] reported that elastogenesis takes place in the shaft of long bones during foetal life, and accumulations of DS by fibroblasts induces the functional deficiency in the elastin-binding protein and, consequently, leads to disruption of normal elastogenesis. Presence of elastic fibers in the limb buds and their primitive perichondrial tissue has been suggested as a crucial factor in maintaining the proper shape of the normal embryonal skeleton. DS damage to tropoelastin could be relevant to skeletal deformations found in MPS disease.. Melbouci hypothesise that MPS IVA is the most severely affected because C6S and KS are GAGs that are accumulated, both of which directly impact bone growth. KS is localised mainly in connective tissue, and C6S is primarily in articular cartilage. Cumulations of these GAGs have a degenerative impact on bones and lead to skeletal deformities [11]. In MPS III, the only GAG that is accumulated is HS. HS is localised in the plasma membrane, the extracellular matrix in visceral organs and the central nervous system [11]. In this study, patients with MPS III showed better growth compared to other MPS types, perhaps because they do not accumulate dermatan sulphate. DS is accumulated in MPS I, MPS II and MPS VI. The differences in growth dynamics between patients with MPS I, MPS II and MPS VI could be explain by various levels of accumulation of toxic DS [11]. Although DS is the GAG accumulated in MPS VI, the upward trend is not intensive, and after the 5th year of life, the growth rate is fairly constant. Growth in MPS VI is related to the severity of the disease. Classification systems describe MPS VI as severe (with early onset of symptoms and fast progression), attenuated (with later onset, slower disease progression and variable clinical presentation) and intermediate; however, MPS VI spans a continuum of disease [25]. The relatively attenuated disease form is characterised by a later onset of symptoms due to lower levels of dermatan sulphate [26]. A study indicates a mild form of MPS VI due to a local East European mutation—p.R152W—in the homozygous state that seems to significantly differ from other MPS VI types [27].

The GAGs that accumulate in MPS I and MPS II are DS and HS. Therefore, it appears that the explanation of growth pattern differences between MPS I and MPS II should be sought in the levels of DS accumulation. MPS I patients show a higher accumulation of DS than MPS II. At the same time, MPS II patients show a higher accumulation of HS than MPS I [28, 29].

We also found a tendency for relationships between growth and the severity of the disease with more severe phenotypes resulting in cognitive impairement. For boys with Hurler syndrome, the body height below the 3rd percentile was reached after the 24th month of life, for patients with severe MPS II between the 6th and 7th year of life, and for patients with attenuated MPS II between the 8th and 9th year. Range of motion limitations (ROM) in patients with MPS II correlate with patients’ height and are more pronounced in a severe form of MPS II. In patients with cognitive impairment, ROM limitations are greater and impact body height more [30]. With age, the number of GAGs excreted decreases, so their highest quantities are accumulated at a young age when they can affect the growth cartilage and cause the most significant damage; hence, we observe a sharp decline in the growth curve.

There are some limitations of this study. Mucopolysaccharidoses are rare diseases, and we only had a limited data sample. Our study has the mix-longitudinal character this method can be used when there is an insufficient number of subjects [31, 32], but non-uniform longitudinal research resulted in a different amount of data for calendar age groups. Therefore, greater reliability of results was obtained for the period, which is better represented.

Although we had a relatively large sample size further studies that would substantiate the observed trends and relationships reported for this group. This could be important for future research that would document changes in growth patterns for children who have access to enzyme replacement therapy. Therefore, every major medical research center should have an anthropologic laboratory with personnel qualified not only to conduct anthropometric measurements but also to accurately interpret the results. Better understanding of the natural history of the disease can be achieved with long-term observations, which help detect and establish patterns of growth and physical development in the evaluated group.

## Conclusions

The long-term follow up showed that:

1. Growth pattern in patients with all types of mucopolysaccharidoses significantly deviates from the general population.
2. Patients with MPS IVA had the most severe growth impairments compared to other patients in the study group.
3. Neuropathic MPS I and II had more severe growth impairments than other patients in this study.
4. Patients with MPS III had the mildest growth impairments compared to other MPS patients and reached the 3rd percentile last.
5. Mean values for MPS patients’ birth length were statistically significantly greater than in the general population. In childhood, growth is good (except MPS IVA), then takes a hit and slows down. The age of exceeding the 3rd percentile is various for different MPS types and depends on disease severity.

## Data Availability

All data generated or analysed during this study are included in this published article.

## Abbreviations

MPS: Mucopolysaccharidoses
GAGs: Glycosaminoglycans
IDUA: Alpha-l-iduronidase
HS: Heparan sulphate
KS: Keratan sulphate
DS: Dermatan sulphate
C6S: Chondroitin sulfate
ROM: Range of motion limitations
